# The Psychological and Academic Effects of Studying From the Home and Host Country During the COVID-19 Pandemic

**DOI:** 10.3389/fpsyg.2021.644096

**Published:** 2021-04-09

**Authors:** Michał Wilczewski, Oleg Gorbaniuk, Paola Giuri

**Affiliations:** ^1^Faculty of Applied Linguistics, Institute of Specialised and Intercultural Communication, University of Warsaw, Warsaw, Poland; ^2^Department of Management, University of Bologna, Bologna, Italy; ^3^Faculty of Social Sciences, Institute of Psychology, John Paul II Catholic University of Lublin, Lublin, Poland; ^4^Faculty of Psychology, University of Economics and Human Sciences in Warsaw, Warsaw, Poland

**Keywords:** COVID-19, quarantine, international students (foreign students), online learning, satisfaction, academic adjustment, acculturative stress, loneliness

## Abstract

**Objective:** This study explored the psychological and academic effects of studying online from the home vis-à-vis host country during the coronavirus disease 2019 (COVID-19) pandemic in the experience of international students at the University of Warsaw, Poland.

**Methods:** A total of 357 international students from 62 countries (236 in the host country and 121 in the home country) completed an online questionnaire survey 2 months after transition to online learning. We studied students' levels of loneliness, life and academic satisfaction, acculturative stress, academic adjustment, performance, loyalty, and perceptions of the online learning experience.

**Results:** The country-of-residence variable had no statistically significant effects on most psychological and academic variables. Significant effects were observed only for two academic variables. Specifically, students who returned to the home country found online communication with other students more contributing to their online learning experience and exhibited higher academic adjustment than students who remained in the host country. This suggests the positive influence of (peer and familial) support on online learning experience from the home country. Furthermore, a significant difference in experiencing acculturative stress occurred for students in quarantine/self-isolation in the host country, which expands prior literature on the disruptive effects of social distancing on students' mental health. Finally, this study confirmed the expected increased levels of loneliness among self-isolating students in both countries, hence extending prior results to the home- and host-country contexts. No relationship between self-isolation and students' life or academic satisfaction was found, which is explained by the specific nature of the learning-from-home experience.

## Introduction

The emergence of the new coronavirus disease 2019 (COVID-19) in December 2019 in Wuhan, China, and a global outbreak of the COVID-19 pandemic in March 2020 have resulted in an unprecedented lockdown of national economies and social distancing restrictions, which had far-reaching economic, political, and social (Bretas and Alon, 2020; Nicola et al., 2020) but also educational consequences. The nationwide closures of educational institutions in 143 countries (UNESCO, 2020) have led to the transition from the conventional to the online learning mode, which dramatically changed studying and working patterns (de Haas et al., 2020).

Although studies show positive working-from-home experiences (e.g., Dubey and Tripathi, 2020), students' experience is generally described as disrupted and leading to feelings of insecurity, anxiety, and hopelessness (Hajdúk et al., 2020; Wang and Zhao, 2020). Students express concerns about economic implications for society, health implications for their families and society, and their own educational and career plans (Cohen et al., 2020). Studies present the pandemic as a disruptive event causing stress that negatively affects students' learning performance and psychological well-being. For example, studies show such adverse effects of lockdowns as increased levels of students' social avoidance (Al-Rabiaah et al., 2020), anxiety (Kaparounaki et al., 2020; Kapasiaa et al., 2020), and a decreased quality of general life (Kaparounaki et al., 2020). Moreover, self-isolating college students suffer from physical and mental health problems (e.g., insomnia, depression) more than those who do not self-isolate (Tang et al., 2020; Zhao et al., 2020; Zhou et al., 2020).

In terms of study experience, students are often dissatisfied with remote learning, as they miss interactions with peers and teachers (de Haas et al., 2020). They perceive their academic experience as difficult and worse than before the pandemic due to the chaotic organization of online learning and a lower quality of online classes as compared to traditional ones (Wilczewski et al., 2020). Moreover, a lack of Internet infrastructure in some underdeveloped locations or unfavorable study conditions in the household prevents students from full engagement in online learning (Kapasiaa et al., 2020).

As compared to domestic students, the negative social and psychological outcomes of the coronavirus outbreak may be more severe for international students who, even in normal conditions, are more prone to mental health problems and do not seek psychological help in the host country (Alharbi and Smith, 2018; Chen et al., 2020). Relocation to another country, leaving family and friends in the home country, entails stress-provoking changes that draw on an individual's coping responses and adjustive resources, which is explained by the stress-and-coping approach to cross-cultural transition (Ward, 2004). Among the stressors that influence students' adjustment and cross-cultural transition are not only situational factors, such as the characteristics of the host country, but also more dynamic factors like the ongoing coronavirus pandemic. As predicted by psychologists, the pandemic-related health crisis and host nationals' fear of the virus led to international students' social exclusion, xenophobic reactions, and an increase in ethnicity and culture-based discrimination (Cohen et al., 2020), especially toward those of Asian background (Rzymski and Nowicki, 2020), which are detrimental to students' mental health, sense of belonging, and self-esteem (King et al., 2020). In turn, the feeling of being perceived negatively by host nationals may affect students' psychological health and hamper their cross-cultural transition, which is reflected in cultural adjustment models (e.g., Kim, 2001). While the negative effects of acculturative stress (e.g., perceived discrimination and communication barrier) decrease the quality of academic experience, adjustment, and mental health (Tummala-Narra et al., 2012), perceived social support mitigates those effects (Ward, 2004; Cura and Işik, 2016; Shu et al., 2020).

The disrupted experience in the host country and campus closures have pushed many international students to travel back to their home country to seek family support. In the USA only, over one million international students returned home in spring 2020 (Dennis, 2020). However, some students had to continue studying in the host country due to closed borders and travel restrictions (Sahu, 2020), being devoid of family and social support structures (Chen et al., 2020).

Despite a growing literature on the disruptive social and emotional outcomes of social distancing and self-isolation on students' psychological well-being, little is known about the impact of the host- and home-country context on the academic experience of international students. Responding to the latest calls for exploring the effects of university closures on students' experiences (Nicola et al., 2020), we will address the following research question: *Is there a difference between international students studying from the home country and those studying from the host country with respect to the psychological and academic effects of online learning during the COVID-19 pandemic?*

Accordingly, this research aimed to explore the psychological and academic effects of studying from the home and host country during the COVID-19 pandemic, based on the experience of international students in the University of Warsaw (UW), Poland; UW is the top 1 and largest Polish and Central European university that switched most teaching activities to online mode in mid-March 2020. Based on prior research on mental health issues of international students and the positive role of social support in their experience in the host country, we assumed that students who participated in online learning from the home country should have better access to support structures (e.g., family, friends) than their counterparts in the host country. Therefore, in such a disruptive experience as learning from home during the pandemic, we hypothesized:

*Hypotheses 1a–c:* Students in the home country show overall (1a) more satisfactory life and (1b) more satisfactory academic experience, as well as (1c) lower perceived discrimination and communication stress than students in the host country.

Moreover, based on prior research on the negative impact of social distancing on students' mental health, it is tentatively hypothesized that, regardless of the country of residence,

*Hypotheses 2a–d:* Students in self-isolation experience (2a) higher perceived acculturative stress, (2b) higher levels of loneliness, (2c) lower life satisfaction, and (2d) lower academic satisfaction than students who do not self-isolate.

## Method

### Research Design

To test the above hypotheses and answer the research question, we conducted a quantitative study that compared the levels of several psychological and academic factors in two groups of international students.

### Sample

As shown in Table 1, 357 international students participated in the research, which comprises 6.5% of all international students at UW. They originated from 62 countries, with the majority coming from Ukraine (21.8%), Belarus (18.7%), China, Spain (5.5% each), Italy (5%), Turkey (4.2%), Russia (2.9%), France (2.4%), and others (under 2%). The number of students who stayed in the host country was nearly twice the number of students who returned home. The majority of participants were female, with gender distribution relatively similar for the host country (female/male = 2.05/1) and home country (female/male = 2.45/1). In both locations, most participants were long-term students, mostly in BA and MA programs. Students in the host country were slightly older as they had an average age of 23.39 years (SD = 4.99) as compared to students who returned home, with an average age of 21.88 years (SD = 4.17). This may be explained by a higher number of MA and Ph.D. students in the host country. Around 60% of students stayed in quarantine or self-isolation when completing the survey. The instruction specified that staying in quarantine/self-isolation referred to “staying at home, going out only for shopping groceries and medicine, medical needs, providing care or help to a vulnerable person, traveling to and from work (where working from home is not possible).”

**Table 1:** Participants' characteristics (*N* = 357).

**Background variables**	**Students in host country (Poland)** **(*N* = 236)**	**Students in home country (*N* = 121)**
Gender		
• Male	77 (32.6%)	35 (28.94%)
• Female	158 (66.9%)	86 (71.1%)
• Other	1 (0.4%)	0
Type of student		
• Long-term	132 (55.9%)	84 (69.4%)
• Short-term (e.g., Erasmus)	104 (44.1%)	37 (33.3%)
Program^a^		
• BA	133 (55.6%)	87 (71.9%)
• MA	85 (36.0%)	29 (24.0%)
• Doctoral	16 (6.8%)	3 (2.5%)
• Other	6 (2.5%)	3 (2.5%)
Staying in quarantine/self-isolation		
• Yes	145 (61.4%)	72 (59.5%)
• No	91 (38.6%)	49 (40.5%)

a *Percentage totals for the program are higher than 100% as some students attended different programs at the same time*.

### Procedure

We collected quantitative data from international students recruited through an email invitation sent out to the whole population of international students at UW (5,500 students) from the university's Welcome Point UW office. Participation was voluntary, and no remuneration was provided. We asked participants to complete an anonymous online questionnaire concerning their online learning experience at UW during the coronavirus pandemic. Apart from the English-language version of the survey, we prepared a Polish version so that international students from Eastern European countries who had a better command of Polish than English could also participate in the study. We back-translated (Brislin, 1970) all scales that have no validated Polish versions in the literature. Because all UW foreign students have a command of English (in international programs) or Polish (in Polish-taught programs) at least at the B2 level, which is verified in the admission process, their language proficiency was sufficient for understanding the questionnaire. In our sample, 126 students (35.3%) chose the Polish version. After giving written consent, they completed the survey over 10 days at the turn of May and June 2020 during the lockdown period in Poland. It took them ~15 min.

### Measures

#### Social and Emotional Loneliness

Students completed the 11-item De Jong Gierveld Loneliness Scale (DJGLS) (de Jong-Gierveld and van Tilburg, 1999) or its Polish adaptation by Grygiel et al. (2013). The scale measures *social loneliness* with five items (e.g., “I can call on my friends whenever I need them;” α = 0.75/0.82 for English/Polish version) and *emotional loneliness* with six items (e.g., “I miss the pleasure of the company of others;” α = 0.79/0.83 for English/Polish version), rated on a 5-point scale (1 = absolutely yes, 5 = absolutely no).

#### Life and Academic Satisfaction

Students rated their satisfaction with general life and academic satisfaction using a 7-point scale (1 = strongly disagree, 7 = strongly agree). They completed the 5-item Satisfaction with Life Scale (SWLS) (Diener et al., 1985) or its Polish adaptation by Jankowski (2015). *Life satisfaction* measures the perception of the general well-being (e.g., “In most ways my life is close to my ideal;” α = 0.79/0.83 for English/Polish version). *Academic satisfaction* was measured with six items capturing satisfaction with (1) general academic experience, (2) studying in the university during the pandemic, (3) studying conditions, (4) online learning in the university, (5) self-perceived scholarly development, and (6) achievement in online learning. Those components constituted a one-factor scale (α = 0.83/0.82 for English/Polish version).

#### Acculturative Stress

Students completed two subscales of the Acculturative Stress Scale (Bashir and Khalid, 2020), rating items on a 5-point scale (1 = strongly disagree, 5 = strongly agree). The first subscale measures *discrimination stress* (i.e., a feeling of unfair treatment) on the grounds of one's international student status, ethnicity, and gender (three items, e.g., “I feel that because I'm a foreign student I'm denied the privileges enjoyed by the local students here;” α = 0.68/0.72 for English/Polish version). The second subscale measures *communication stress* (three items, e.g., “I feel difficulty communicating with local people due to the language barrier;” α = 0.75/0.72 for English/Polish version).

#### Online Learning Experience, Academic Adjustment, Performance, and Loyalty

All academic variables were measured using a 7-point scale (1 = strongly disagree, 7 = strongly agree). The online learning experience was captured with a self-developed 12-item scale measuring four aspects (3 items per scale) of online learning: (1) *interactions with students* (e.g., “Communication with other students has had a positive effect on my online learning experience;” α = 0.75/0.72 for English/Polish version); (2) *interactions with teachers* (e.g., “I find my teachers supportive in my online learning;” α = 0.78/0.80 for English/Polish version); (3) *technical capacity* (e.g., “I have enough technical ability to participate in online classes;” α = 0.76/0.66 for English/Polish version); and (4) *organization of online learning* (e.g., “My university organizes online learning well;” α = 0.81/0.75 for English/Polish version).

*Academic adjustment* (i.e., the degree to which a student fits in the academic context and how comfortable they feel in that context) was measured with five items expressing adjustment to (1) teaching methods, (2) student assessment methods, (3) teachers' expectations toward students, (4) studying conditions, and (5) online learning. The scale yielded a one-factor structure (α = 0.83 for both English and Polish version).

*Academic performance* and *student loyalty* were measured with one item per variable, respectively, “My academic performance is better in online learning than before the COVID-19 pandemic” and “I would not recommend online learning experience in this university to other students” (reverse coded), similarly to prior studies (e.g., van Rooij et al., 2018).

#### Control Variables

We included *age* and *personality traits* as control variables (see Table 2) to check potential differences between students in the host and home country. Students completed the Ten-Item Personality Inventory (TIPI) (Gosling et al., 2003), which measures the Big Five dimensions; a Polish version developed by Sorokowska et al. (2014) was used. Students rated, on a 7-point scale (1 = disagree strongly, 7 = agree strongly), the extent to which five pairs of traits applied to them (e.g., “I see myself as extraverted, enthusiastic”). The traits involved (Cronbach's alpha for English/Polish version): extraversion (0.68/0.70), agreeableness (0.40/0.50), conscientiousness (0.50/0.76), emotional stability (0.73/0.65), and openness to experiences (0.45/0.47).

**Table 2:** Age and personality differences between students staying in the host (*N* = 236) vs. home (*N* = 121) country.

**Control variables**	**Country**	***M***	***SD***	***t*_**(356)**_**
Age	Host	23.39	4.99	2.87^**^
	Home	21.88	4.17	
Personality traits				
• Extraversion	Host	4.66	1.46	−0.70
	Home	4.78	1.54	
• Agreeableness	Host	5.10	1.23	0.75
	Home	5.00	1.23	
• Conscientiousness	Host	5.27	1.44	−0.57
	Home	5.36	1.42	
• Emotional stability	Host	4.47	1.56	1.24
	Home	4.25	1.62	
• Openness to experiences	Host	5.17	1.20	−0.31
	Home	5.21	1.04	

** *p< 0.01*.

### Data Analysis

We performed descriptive statistics, *t*-test, and 1-ANCOVA to compare groups of students, applying age and personality traits as covariates (control variables). The sample allowed us to identify differences between the measured variables (see Table 3) between students in the host vs. home country with the effect size of at least Cohen's *d* = 0.30 and probability of 1 – β = 0.90 for two-tailed tests and 1 – β = 0.80 for one-tailed tests with significance at α = 0.05.

**Table 3:** The effects of staying in the host (*N* = 236) vs. home (*N* = 121) country and staying vs. not staying in quarantine/self-isolation on international students' well-being and academic experience^a^.

**Variable**					**Staying in quarantine/self-isolation**					
		**Staying in host country**^**b**^			**Students in host country**^**c**^			**Students in home country**^**d**^		
		***M***	***SD***	***F*_**(1, 349)**_**	***M***	***SD***	***F*_**(1, 228)**_**	***M***	***SD***	***F*_**(1, 113)**_**
Social loneliness	Yes	2.57	0.85	2.21	2.66	0.84	3.13^#^	2.52	0.88	2.47
	No	2.42	0.87		2.43	0.87		2.28	0.83	
Emotional loneliness	Yes	3.06	0.88	1.85	3.16	0.83	3.16^#^	3.04	0.91	3.37^#^
	No	2.94	0.91		2.90	0.94		2.78	0.89	
Life satisfaction	Yes	4.50	1.13	0.00	4.41	1.14	1.77	4.47	1.27	0.10
	No	4.49	1.30		4.64	1.11		4.51	1.37	
Academic satisfaction	Yes	4.45	1.22	0.44	4.46	1.22	0.00	4.46	1.17	0.21
	No	4.49	1.21		4.44	1.22		4.53	1.28	
Discrimination stress	Yes	2.06	0.78	2.39	2.19	0.79	9.14^##^	2.05	0.74	4.49^##^
	No	1.94	0.73		1.86	0.74		1.78	0.68	
Communication stress	Yes	2.99	1.13	0.36	3.09	1.09	1.74	2.88	1.08	2.38
	No	3.01	1.07		2.83	1.18		3.21	1.04	
Interactions with students	Yes	3.88	1.43	5.57^*^	3.88	1.43	0.04	4.33	1.37	0.26
	No	4.26	1.49		3.87	1.42		4.17	1.67	
Interactions with teachers	Yes	4.49	1.39	0.30	4.49	1.45	0.04	4.50	1.53	0.00
	No	4.50	1.52		4.49	1.31		4.50	1.52	
Student's technical capacity	Yes	5.79	1.20	1.09	5.77	1.31	0.00	5.61	1.14	0.09
	No	5.64	1.10		5.83	1.02		5.68	1.06	
Organization of learning	Yes	4.46	1.35	1.81	4.48	1.35	0.14	4.56	1.33	0.34
	No	4.61	1.34		4.42	1.35		4.69	1.36	
Academic adjustment	Yes	4.95	1.20	4.01^*^	4.94	1.18	0.02	5.09	1.09	0.96
	No	5.17	1.00		4.96	1.23		5.28	0.85	
Academic performance	Yes	3.09	1.66	0.17	3.14	1.68	0.22	3.10	1.67	0.48
	No	3.19	1.68		3.02	1.63		3.33	1.70	
Student loyalty	Yes	4.45	1.87	0.48	4.44	1.94	0.05	4.15	1.70	0.54
	No	4.24	1.91		4.46	1.77		4.37	2.21	

a *Covariates: age and personality traits*.

* *p< 0.05 for two-tailed tests*.

# *p < 0.05*,

## *p < 0.01 for one-tailed tests*.

b *Comparisons between students staying in the host (Yes) and home (No) country during the pandemic*.

c *Comparisons between students staying (Yes) and not staying (No) in quarantine/self-isolation in the host country during the pandemic*.

d *Comparisons between students staying (Yes) and not staying (No) in quarantine/self-isolation in the home country during the pandemic*.

## Results

First, we checked if students' decision making concerning turning back to the home country was related to their age or personality profile (see Table 2). The analysis showed statistically significant differences in age [*t*(356) = 2.87, *p* < 0.01] in that older students tended to stay in the host country. No significant differences between students in the host and home country were found for any personality trait within the Big Five model.

As shown in Table 3, statistically significant differences between students staying in the host and home country during the pandemic were found in their online communication with other students [*F*(1, 349) = 5.57, *p* < 0.05; Cohen's *d* = 0.26]. Students in the home country found online communication with other students more important in their online learning experience than those in the host country. Moreover, students in their home country showed higher academic adjustment than those in the host country [*F*(1,364) = 4.01, *p* < 0.05; Cohen's *d* = 0.20].

No country-related differences were found for experiencing acculturative stress, neither discrimination [*F*(1, 349) = 2.39, *p* = n.s.] nor communication stress [*F*(1, 349) = 0.36, *p* = n.s.]. However, students who stayed in self-isolation exhibited higher discrimination stress than their counterparts who did not self-isolate, both in the host country [*F*(1, 228) = 9.14, *p* < 0.01; Cohen's *d* = 0.39] and home country [*F*(1, 113) = 4.49, *p* < 0.01, Cohen's *d* = 0.38].

Finally, students in self-isolation in the host country showed higher social [*F*(1, 228) = 3.13, *p* < 0.05; Cohen's *d* = 0.27] and emotional loneliness [*F*(1, 228) = 3.16, *p* < 0.05; Cohen's *d* = 0.29] than their counterparts who did not self-isolate. The same direction of difference was found for students in quarantine/self-isolation in their home country for emotional loneliness [*F*(1, 113) = 3.37, *p* < 0.05; Cohen's *d* = 0.29].

No statistically significant differences were found for experiencing life and academic satisfaction for students in self-isolation and those who did not self-isolate (see Table 3).

## Discussion

The outbreak of the COVID-19 pandemic has caused an unprecedented health crisis that disrupted education institutions globally by pushing online activities to the online mode. Campus closures and social distancing measures introduced to stop the spread of the virus deprived students of the socializing aspect, negatively impacting their mental health. The pandemic experience was particularly challenging for international students who were devoid of adequate social support (family, friends) their domestic peers had. Furthermore, international students often struggled with immigration issues (Chirikov and Soria, 2020) and visa extension issues due to traveling restrictions (Hope, 2020; Wilczewski et al., 2020). In turn, those who managed to turn back to their home country could not fully enjoy the studying conditions agreed on in the pre-pandemic contract.

This research explored the psychological and academic effects of studying from the home and host country during the COVID-19 pandemic, using online survey data from international students in UW, Poland. By developing our understanding of international students' life and academic experience, we make several contributions to the psychological and international higher education literature. First, results showed that the country-of-residence variable was not significantly correlated with psychological variables (Hypothesis 1a rejected) but only with two aspects of academic experience (Hypothesis 1b partly supported). Specifically, students in their home country found communication with peers more contributing to their online learning experience and showed higher adjustment. This result is in line with cultural adjustment frameworks (Black et al., 1991; Kim, 2001; Ward, 2004) that view communication competence (with an individual's affective, cognitive, and behavioral skills) and communication interactions as critical determinants of adjustment. Moreover, they link adjustment with (familial, peer) support structures, which is supported by academic adjustment research (e.g., Shu et al., 2020). The result implies that universities should pay careful attention to fostering peer communication in online learning (e.g., by promoting group work and collaborative learning) and developing social support structures (e.g., by fostering online discussion groups allowing students to share experiences with peers, or online social activities attenuating the sense of loneliness), as these enhance academic adjustment. Moreover, because students in the home country show higher levels of academic adjustment than students in the host country, universities should develop compensatory support services for the latter based on their unique nationalities and cultures (Chirikov and Soria, 2020), which could enhance their adjustment to online learning.

We found no support for the hypothesized country-specific differences in the experience of acculturative stress (Hypothesis 1c rejected). The lack of country-specific differences in the perceived discrimination and communication stress may result from the nature of the learning-from-home experience itself. This experience considerably limits social interactions with other students and locals, thereby limiting occasions for comparing international students' experience to other students' experience.

However, a difference in experiencing discrimination stress occurs for students in self-isolation in the host and home country; no such difference was found for communication stress, which may be explained by social distancing and limited opportunities for communication with locals in the host country (Hypothesis 2a partly supported). A higher level of perceived discrimination resonates with prior research on the negative effect of social distancing on students' mental health (Fu et al., 2020; Tang et al., 2020; Zhao et al., 2020). It may suggest that the disruptive experience of social isolation may arouse the feeling of receiving lower-quality service as compared to other students, which may be caused by the fundamental attribution error (Ross, 1977). Self-isolation is a stressor that causes boredom and increased emotional distress and dissatisfaction (Presti et al., 2020; Yan et al., 2021). Moreover, besides learning from home, self-isolation potentially exposes students to smartphone/Internet overuse that has been linked with depressiveness (Duan et al., 2020). Accordingly, self-isolating students may attribute their dissatisfaction with the disrupted experience to the university. Thus, to minimize the negative implications of self-isolation for international students, universities should pay careful attention to more effective and culture-sensitive communication of pandemic-related messages and support structures available at the university. For example, email messages informing students on the organization of online learning, virtual events, and other services (e.g., extracurricular courses, psychological help) could be sent out to all students in both the host language and English, so that international students are aware of their fair treatment as compared to domestic students. Moreover, to enhance students' coping responses to the pandemic, students in quarantine or self-isolation should be provided with special psychological support and therapy through digital technology (Xiang et al., 2020), especially given the fact that international students highly appreciate psychological help received from the university and expect an increased number of therapeutic sessions (Wilczewski et al., 2020). Notably, training students on stress management could also contribute to their coping with emotional distress (Yan et al., 2021) and satisfaction with university services.

Finally, the expected increased levels of social and emotional loneliness among self-isolating students in both countries were essentially confirmed in this study, with the difference in experiencing social loneliness in the home country being close to reaching statistical significance (Hypothesis 2b partly supported). Our results resonate with the results of prior research on domestic students' mental health and loneliness (Elmer et al., 2020) and extend their validity to international students studying in the host and home country. However, no statistically significant relation of self-isolation with a student's life or academic satisfaction was found (Hypotheses 2c and 2d rejected, respectively), which may again be explained by the specific nature of the academic experience of both groups of students that participated in learning from home no matter if in self-isolation or not.

This study is not without limitations that, nevertheless, warrant avenues for future research. First, the cross-sectional nature of this study prevented us from determining how long the benefits/challenges related to participation in online learning from the home or host country would last. Although longitudinal research could resolve this important question, we are aware that researchers have limited opportunities to reach out to students with similar research instruments during the COVID-19 pandemic when students are systematically invited by the university to participate in online surveys to monitor how they perform in online learning. To overcome this limitation, universities could develop research policies promoting collaboration between researchers and the transfer of knowledge across research projects drawing on data collected from students. Second, due to the limited space in the questionnaire survey, this research did not measure other psychological factors that could explain country-specific differences in students' mental health. As the most recent research has found the critical role of coping styles and the dark triad in coping during the pandemic (e.g., Nowak et al., 2020; Zajenkowski et al., 2020; Triberti et al., 2021; Yan et al., 2021), those should be considered as control variables in future research on the international student experience in the host vs. home country. Third, although we captured student online learning experience, we did not measure students' support structures (e.g., perceptions of contacts with family, peers, or social networking). Involving those variables could explain the observed differences in the perceived role of online communication and acculturative stress in a more comprehensive way. Finally, future qualitative studies could better contextualize research into students' learning experiences from the host and home country, thereby exposing country-specific factors (e.g., different social distancing patterns) determining students' life and academic experiences.

## Data Availability Statement

The raw data supporting the conclusions of this article will be made available by the authors, without undue reservation.

## Ethics Statement

The studies involving human participants were reviewed and approved by The Rector's Committee for Ethics of Research with Human Participants at the University of Warsaw. The patients/participants provided their written informed consent to participate in this study.

## Author Contributions

MW planned the study, developed instruments, conducted the study, and drafted the manuscript. OG revised instruments, conducted statistical analyses, and participated in the interpretation of data and writing up results. PG supervised all the processes, provided critical guidance, participated in data interpretation, and revised the manuscript. All authors contributed to the article and approved the submitted version.

## Conflict of Interest

The authors declare that the research was conducted in the absence of any commercial or financial relationships that could be construed as a potential conflict of interest.

## References

[B1] Alharbi E. S. Smith A. P. (2018). Review of the literature on stress and wellbeing of international students in English-speaking countries. Int. Educ. Stud. 11:22. 10.5539/ies.v11n6p22

[B2] Al-Rabiaah A. Temsah M. H. Al-Eyadhy A. A. Hasan G. M. Al-Zamil F. Al-Subaie S.. (2020). Middle East Respiratory Syndrome-Corona Virus (MERS-CoV) associated stress among medical students at a university teaching hospital in Saudi Arabia. J. Infect. Public Health 13, 687–691. 10.1016/j.jiph.2020.01.005 32001194 PMC7102651

[B3] Bashir A. Khalid R. (2020). Development and validation of the acculturative stress scale for Pakistani Muslim students. Cogent Psychol. 7:1714101. 10.1080/23311908.2020.1714101

[B4] Black J. S. Mendenhall M. E. Oddou G. (1991). Toward a comprehensive model of international adjustment: an integration of multiple theoretical perspectives. Acad. Manag. Rev. 16, 291–317. 10.5465/amr.1991.4278938

[B5] Bretas V. P. G. Alon I. (2020). The impact of COVID-19 on franchising in emerging markets: an example from Brazil. Glob. Bus. Organ. Excell. 39, 6–16. 10.1002/joe.22053

[B6] Brislin R. W. (1970). Back-translation for cross-cultural research. J. Cross Cult. Psychol. 1, 185–216. 10.1177/135910457000100301

[B7] Chen J. H. Li Y. Wu A. M. S. Tong K. K. (2020). The overlooked minority: mental health of International students worldwide under the COVID-19 pandemic and beyond. Asian J. Psychiat. 54, 1–2. 10.1016/j.ajp.2020.102333 32795955 PMC7399745

[B8] Chirikov I. Soria K. M. (2020). “International students' experiences and concerns during the pandemic,” in SERU Consortium, University of California–Berkeley and University of Minnesota. Available online at: https://escholarship.org/uc/item/43q5g2c9 (accessed December 12, 2020).

[B9] Cohen A. K. Hoyt L. T. Dull B. (2020). A descriptive study of COVID-19–related experiences and perspectives of a national sample of college students in spring 2020. J. Adolesc. Health 67, 369–375. 10.1016/j.jadohealth.2020.06.009 32593564 PMC7313499

[B10] Cura Ü. Işik A. N. (2016). Impact of acculturative stress and social support on academic adjustment of international students. Egitim Bilim 41, 333–347. 10.15390/EB.2016.6158

[B11] de Haas M. Faber R. Hamersma M. (2020). How COVID-19 and the Dutch “intelligent lockdown” change activities, work, and travel behaviour: evidence from longitudinal data in the Netherlands. Transp. Res. Interdiscip. Perspect. 6, 1–11. 10.1016/j.trip.2020.100150 PMC7284275 34171019

[B12] de Jong-Gierveld J. van Tilburg T. G. (1999). Manual of the Loneliness Scale. Available online at: http://home.fsw.vu.nl/tg.van.tilburg/manual_loneliness_scale_1999.html (accessed December 12, 2020).

[B13] Dennis M. J. (2020). COVID-19 will accelerate the decline in international student enrollment. Enroll. Manag. Rep. 24, 1–5. 10.1002/emt.30672

[B14] Diener E. Emmons R. Larsen J. Griffin S. (1985). The satisfaction with life scale. J. Pers. Assess. 49, 71–75. 10.1207/s15327752jpa4901_13 16367493

[B15] Duan L. Shao X. Wang Y. Huang Y. Miao J. Yang X.. (2020). An investigation of mental health status of children and adolescents in china during the outbreak of COVID-19. J. Affect. Disord. 275, 112–118. 10.1016/j.jad.2020.06.029 32658812 PMC7329661

[B16] Dubey A. D. Tripathi S. (2020). Analysing the Sentiments towards work-from-home experience during COVID-19 Pandemic. J. Innov. Manag. 8, 13–19. 10.24840/2183-0606_008.001_0003

[B17] Elmer T. Mepham K. Stadtfeld C. (2020). Students under lockdown: Comparisons of students' social networks and mental health before and during the COVID-19 crisis in Switzerland. PLoS ONE 15:e0236337. 10.1371/journal.pone.0236337 32702065 PMC7377438

[B18] Fu W. Wang C. Zou L. Guo Y. Lu Z. Yan S.. (2020). Psychological health, sleep quality, and coping styles to stress facing the COVID-19 in Wuhan, China. Transl. Psychiatry 10, 1–9. 10.1038/s41398-020-00913-3 32647160 PMC7347261

[B19] Gosling S. D. Rentfrow P. J. Swann W. B. (2003). A very brief measure of the Big-Five personality domains. J. Res. Pers. 37, 504–528. 10.1016/S0092-6566(03)00046-128810502

[B20] Grygiel P. Humenny G. Rebisz S. Kwitaj P. Sikorska J. (2013). Validating the polish adaptation of the 11-item de Jong Gierveld Loneliness Scale. Eur. J. Psychol. Assess. 29, 129–139. 10.1027/1015-5759/a000130

[B21] Hajdúk M. Dančík D. Januška J. Svetský V. Straková A. Turček M.. (2020). Psychotic experiences in student population during the COVID-19 pandemic. Schizophr. Res. 222, 520–521. 10.1016/j.schres.2020.05.023 32405153 PMC7218396

[B22] Hope J. (2020). Be aware of how COVID-19 could impact international students. Success. Regist. 20, 1–8. 10.1002/tsr.3070833712081

[B23] Jankowski K. S. (2015). Is the shift in chronotype associated with an alteration in well-being? Biol. Rhythm Res. 46, 237–248. 10.1080/09291016.2014.985000

[B24] Kaparounaki C. K. Patsali M. E. Mousa D. P. V. Papadopoulou E. V. K. Papadopoulou K. K. K. Fountoulakis K. N. (2020). University students' mental health amidst the COVID-19 quarantine in Greece. Psychiatry Res. 290, 1–2. 10.1016/j.psychres.2020.113111 32450416 PMC7236729

[B25] Kapasiaa N. Paulb P. Royc A. Sahac J. Zaveric A. Mallickc R.. (2020). Impact of lockdown on learning status of undergraduate and postgraduate students during COVID-19 pandemic in West Bengal, India. Child. Youth Serv. Rev. 116, 1–5. 10.1016/j.childyouth.2020.105194 32834270 PMC7308748

[B26] Kim Y. Y. (2001). Becoming Intercultural: An Integrative Theory of Communication and Cross-Cultural Adaptation. Thousand Oaks, CA: Sage.

[B27] King J. A. Cabarkapa S. Leow F. H. P. Ng C. H. (2020). Addressing international student mental health during COVID-19: an imperative overdue. Aust. Psychiatry 28:469. 10.1177/1039856220926934 32427498

[B28] Nicola M. Alsafi Z. Sohrabi C. Kerwan A. Al-Jabir A. Iosifidis C.. (2020). The socio-economic implications of the coronavirus pandemic (COVID-19): a review. Int. J. Surg. 78, 185–193. 10.1016/j.ijsu.2020.04.018 32305533 PMC7162753

[B29] Nowak B. Brzóska P. Piotrowski J. Sedikides C. Zemojtel-Piotrowska M. Jonason P. K. (2020). Adaptive and maladaptive behavior during the COVID-19 pandemic: the roles of Dark Triad traits, collective narcissism, and health beliefs. Pers. Individ. Differ. 167:110232. 10.1016/j.paid.2020.110232 32834282 PMC7363424

[B30] Presti G. Mchugh L. Gloster A. Karekla M. Haye S. C. (2020). The dynamics of fear at the time of COVID-19: a contextual behavioral science perspective. Clin. Neuropsychiatry 17, 65–71. 10.36131/CN20200206 PMC8629087 34908970

[B31] Ross L. (1977). The intuitive psychologist and his shortcomings: distortions in the attribution process. Adv. Exp. Soc. Psychol. 10, 173–220. 10.1016/S0065-2601(08)60357-3

[B32] Rzymski P. Nowicki M. (2020). COVID-19-related prejudice toward Asian medical students: a consequence of SARS-CoV-2 fears in Poland. J. Infect. Public Health 13, 873–876. 10.1016/j.jiph.2020.04.013 32387102 PMC7196414

[B33] Sahu P. (2020). Closure of universities due to coronavirus disease 2019 (COVID-19): impact on education and mental health of students and academic staff. Cureus 12:e7541. 10.7759/cureus.7541 32377489 PMC7198094

[B34] Shu F. Ahmed S. F. Pickett M. L. Ayman R. McAbee S. T. (2020). Social support perceptions, network characteristics, and international student adjustment. Int. J. Intercult. Relat. 74, 136–148. 10.1016/j.ijintrel.2019.11.002

[B35] Sorokowska A. Słowińska A. Zbieg A. Sorokowski P. (2014). Polska adaptacja testu Ten Item Personality Inventory (TIPI)—TIPI-PL—wersja standardowa i internetowa. Wrocław: WrocLab.

[B36] Tang W. Hu T. Yang L. Xu J. (2020). The role of alexithymia in the mental health problems of home-quarantined university students during the COVID-19 pandemic in China. Pers. Individ. Differ. 165, 1–7. 10.1016/j.paid.2020.110131 32518435 PMC7273169

[B37] Triberti S. Durosini I. Pravettoni G. (2021). Social distancing is the right thing to do: dark triad behavioral correlates in the COVID-19 quarantine. Pers. Individ. Differ. 170:110453. 10.1016/j.paid.2020.110453

[B38] Tummala-Narra P. Alegria M. Chen C. N. (2012). Perceived discrimination, acculturative stress, and depression among South Asians: mixed findings. Asian Am. J. Psychol. 3, 3–16. 10.1037/a0024661

[B39] UNESCO (2020). Education: From Disruption to Recovery. Available online at: https://en.unesco.org/covid19/educationresponse (accessed December 12, 2020).

[B40] van Rooij E. C. M. Jansen E. P. W. A. van de Grift W. J. C. M. (2018). First-year university students' academic success: the importance of academic adjustment. Eur. J. Psychol. Educ. 33, 749–767. 10.1007/s10212-017-0347-8

[B41] Wang C. Zhao H. (2020). The Impact of COVID-19 on anxiety in Chinese university students. Front. Psychol. 11:1168. 10.3389/fpsyg.2020.01168 32574244 PMC7259378

[B42] Ward C. (2004). “Psychological theories of culture contact and their implications for intercultural training and interventions,” in Handbook of Intercultural Training, eds Landis D. Bennett J. Bennett M. J. (https://www.google.com/search?sxsrf=ALeKk02ZkFwueh9Pe002Pfa1VrECh8jC1A:1614876121401&q=Thousand+Oaks,+California&stick=H4sIAAAAAAAAAOPgE-LUz9U3sEw2MC9R4gAxiyySLbSMMsqt9JPzc3JSk0sy8_P084vSE_MyqxJBnGKrjNTElMLSxKKS1KJihZz8ZLDwIlbJkIz80uLEvBQF_8TsYh0F58SczLT8orzMxB2sjABqF7C_agAAAA&sa=X&ved=2ahUKEwjC28msipfvAhUXxzgGHTjoAZMQmxMoATAqegQINRAD Thousand Oaks, CA: Sage), 185–216.

[B43] Wilczewski M. Gorbaniuk O. Mughan T. Wilczewska E. (2020). “Our Academic Life has Simply Died.” Online Learning Experience During the Covid-19 Pandemic: Evidence from Poland. [Unpublished article].

[B44] Xiang Y. T. Yang Y. Li W. Zhang L. Zhang Q. Cheung T.. (2020). Timely mental health care for the 2019 novel coronavirus outbreak is urgently needed. Lancet Psychiatry 7, 228–229. 10.1016/S2215-0366(20)30046-8 32032543 PMC7128153

[B45] Yan L. Gan Y. Ding X. Wu J. Duan H. (2021). The relationship between perceived stress and emotional distress during the COVID-19 outbreak: effects of boredom proneness and coping style. J. Anxiety Disord. 77:102328. 10.1016/j.janxdis.2020.102328 33160275 PMC7598556

[B46] Zajenkowski M. Jonason P. K. Leniarska M. Kozakiewicz Z. (2020). Who complies with the restrictions to reduce the spread of COVID-19?: personality and perceptions of the COVID-19 situation. Pers. Individ. Differ. 166:110199. 10.1016/j.paid.2020.110199 32565591 PMC7296320

[B47] Zhao Y. An Y. Tan X. Li X. (2020). Mental health and its influencing factors among self-isolating ordinary citizens during the beginning epidemic of COVID-19. J. Loss Trauma 25, 580–593. 10.1080/15325024.2020.1761592

[B48] Zhou S.-J. Wang L.-L. Yang R. Yang X.-J. Zhang L.-G. Guo Z.-C.. (2020). Sleep problems among Chinese adolescents and young adults during the coronavirus-2019 pandemic. Sleep Med. 74, 39–47. 10.1016/j.sleep.2020.06.001 32836185 PMC7274988

